# Global and local processing near the left and right hands

**DOI:** 10.3389/fpsyg.2013.00793

**Published:** 2013-10-29

**Authors:** Robin M. Langerak, Carina L. La Mantia, Liana E. Brown

**Affiliations:** Department of Psychology, Trent UniversityPeterborough, ON, Canada

**Keywords:** peripersonal space, multisensory integration, visual processing, hemispheric specialization, laterality

## Abstract

Visual targets can be processed more quickly and reliably when a hand is placed near the target. Both unimodal and bimodal representations of hands are largely lateralized to the contralateral hemisphere, and since each hemisphere demonstrates specialized cognitive processing, it is possible that targets appearing near the left hand may be processed differently than targets appearing near the right hand. The purpose of this study was to determine whether visual processing near the left and right hands interacts with hemispheric specialization. We presented hierarchical-letter stimuli (e.g., small characters used as local elements to compose large characters at the global level) near the left or right hands separately and instructed participants to discriminate the presence of target letters (X and O) from non-target letters (T and U) at either the global or local levels as quickly as possible. Targets appeared at either the global or local level of the display, at both levels, or were absent from the display; participants made foot-press responses. When discriminating target presence at the global level, participants responded more quickly to stimuli presented near the left hand than near either the right hand or in the no-hand condition. Hand presence did not influence target discrimination at the local level. Our interpretation is that left-hand presence may help participants discriminate global information, a right hemisphere (RH) process, and that the left hand may influence visual processing in a way that is distinct from the right hand.

## Introduction

A growing body of work demonstrates that people process visual information differently when stimuli are presented near to rather than far from their hands. Neuropsychological studies, on the whole, indicate that placing a stimulus near one of the hands reduces perceptual and attentional impairments. Visual extinction deficits have been reduced by presenting stimuli near-hand in the contralesional visual field, tactile extinction is exacerbated by presenting a visual stimulus near the ipsilesional hand (Ladavas et al., 1998; di Pellegrino and Frassinetti, 2000), and both detection (Schendel and Robertson, 2004) and discrimination (Brown et al., 2008) benefits have been documented in the defective visual field of cortically-blind patients. Studies of healthy undergraduates have shown that placing a target near one hand has typically led to observations of perceptual facilitation. Placing a hand near a visual target speeds target detection (Reed et al., 2006, 2010; Jackson et al., 2010), causes tactile interference (Spence, 2002), speeds the assignment of figure and ground (Cosman and Vecera, 2010), and leads to greater reaching precision in comparison to responses to targets that appear in the same location but without a nearby hand (Brown et al., 2009). Other studies indicate that people are slower to disengage from visual targets when they appear near the hands (Abrams et al., 2008; Thura et al., 2008; Tseng and Bridgeman, 2011), and that nearby hands slow switching between the global and local levels of a stimulus (Davoli et al., 2012). Evidence suggests that these psychophysical effects are stronger in the presence of the participants' real hand than a fake one (Reed et al., 2006; Brown et al., 2009), while others indicate that near-hand effects can be linked to the presence of an avatar-hand whose movements mirror the actions of the participants' real hand but are not linked to an unmoving avatar (Short and Ward, 2009). Together, this evidence suggests that visual stimuli are processed differently when the observer's own hand(s) is placed near the stimulus rather than when the hand is placed elsewhere.

Compatible explanations for near-hand effects have been offered both at cognitive and neural levels. At the cognitive level, explanations associate hand-presence with the mobilization of additional perceptual (e.g., Cosman and Vecera, 2010) or cognitive processing resources [e.g., attention or working memory (e.g., Reed et al., 2006, 2010; Abrams et al., 2008; Tseng and Bridgeman, 2011; Davoli et al., 2012)]. Cognitive-level accounts are consistent with neural-level accounts in that they both propose that the hands bring additional resources to bear on processing nearby targets. At the neural level, explanations for near-hand effects have focused on findings in the monkey neurophysiology literature showing that 3D visual objects presented in the space near the hands and face recruit visual-tactile bimodal neurons. These neurons have tactile receptive fields (tRFs) on the skin and visual receptive fields (vRFs) that include and extend beyond the tRF into the space surrounding the hand or face. They are activated in response to either tactile or visual stimuli presented on or near the skin (Graziano and Gross, 1993; Graziano et al., 1994; Graziano, 1999; Graziano and Gandhi, 2000; Graziano and Cooke, 2006). These neurons code space near the hand and face more robustly than other body parts, and near-hand space is represented more robustly than space far from the hand (Graziano et al., 1994; Graziano, 1999; Graziano and Cooke, 2006).

Functional imaging studies in humans show that targets appearing near a hand selectively activate and cause adaptation in the intraparietal sulcus (IPS; Makin et al., 2007), supramarginal gyrus (SMG), and in both the dorsal and ventral premotor cortex (PMd and PMv, respectively) in comparison to targets appearing far from the hand (Brozzoli et al., 2011). Other work (Gentile et al., 2011) demonstrated that PMv, PMd, and SMG all showed BOLD-signal increases to near-hand unimodal visual and unimodal tactile stimuli, additive responses to bimodal visual-tactile stimuli, and PMd and anterior IPS showed superadditive responses to bimodal stimuli (i.e., the response to bimodal stimuli was greater than predicted from the sum of responses to unimodal stimuli). Together, these studies suggest that near-hand visual targets recruit multisensory neural resources, like bimodal and multimodal cells, and that these effects are similar both in monkeys and humans. This recruitment may allow for a more robust visual representation of the target, and support the processing benefits associated with near-hand space. This explanation can be likened to the facilitation that appears to explain redundancy effects (the finding that humans respond more quickly to two identical stimuli than to one, even when factors like stimulus size and brightness are controlled; Raab, 1962; Gielen et al., 1983). It may be that visual stimuli appearing near a hand recruit additional (multisensory) brain regions for processing that are not recruited in the hand-absent case, and that this additional recruitment influences visual processing. Tests of a computational model using this general principle have been promising (Magosso et al., 2010b).

Given that motor and sensory representations of the hand are lateralized to the contralateral hemisphere both for simple (Bryden, 1982; Graziano, 1999; Jones and Lederman, 2006) and patterned (Reed et al., 2009) stimuli, our study focuses on whether effects near the left and right hands interact in a meaningful way with a task known to differentially tap the left and right hemispheres (RHs). The general nature of hemispheric specialization is relatively well-known. Classically, language is thought to be lateralized to the left hemisphere (LH) while visuospatial judgments are lateralized to the RH (Kimura and Durnford, 1974; Kinsbourne and Hicks, 1978; Bryden, 1982; Bradshaw and Nettleton, 1983; Corballis, 1989). With regard to specific tasks that demonstrate lateralized visual processing, Navon's (1977) hierarchical forms have been used to study differences between global and local processing and their relative lateralization (e.g., see Table 1). In her classic study, Sergent (1982) used hierarchical forms consisting of two target letters, H and L, and two distracter letters, F and S, and asked that participants indicate with a button press whether one of the target letters was present in a stimulus. The target could be present at the global level (the large letter), the local level (the small letter), at both levels, or at neither. Hemispheric specialization was tested by presenting the stimuli either in the left or right visual field, as visual information presented in the left visual field projects to the RH and visual information presented in the right visual field projects to the LH. Sergent found that response latency depended both on target level and visual field. Global-level targets were processed more quickly when the hierarchical figure appeared in the left visual field RH than in the right visual field LH. Conversely, local-level targets were processed more quickly when the hierarchical figure was presented centrally or in the right visual field LH in comparison to the left visual field RH. Sergent (1982) interpreted this pattern as evidence that global information is preferentially processed in the RH and that local information is preferentially processed in the LH.

**Table 1:**
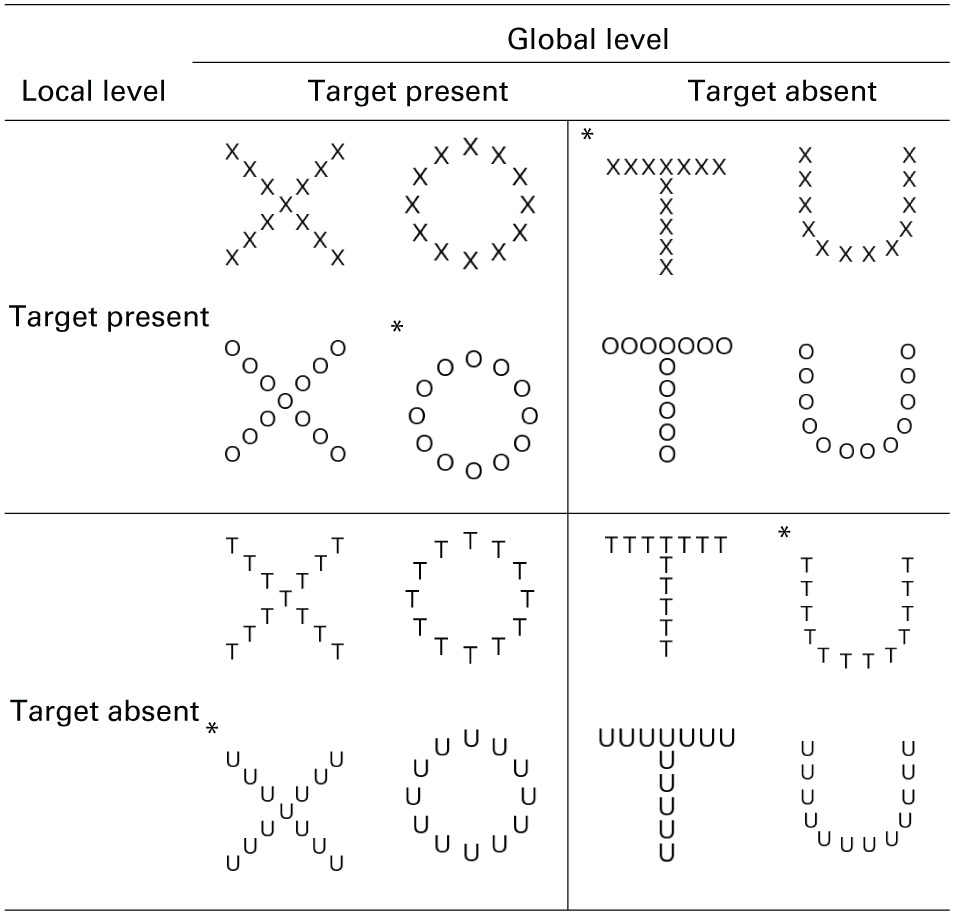
**Experimental stimuli, hierarchical form displays of global target-present (large letters X and O) and global target-absent items (large letters T and U) composed of local target-present (small component Xs and Os) and local target-absent items (small component Ts and U)**.

^*^example stimuli shown during instructions.

Sergent (1982) and other researchers have acknowledged that the distinction between global and local processing may come down to a distinction between visual processing of low and high spatial frequency information, respectively (Shulman et al., 1986; Christman et al., 1991; Kitterle et al., 1992; Flevaris et al., 2010, 2011). In general, these studies associate global/low-spatial-frequency processing with RH function and local/high-spatial-frequency with LH function (Karim and Kojima, 2010). This lateralization pattern has been supported by studies of neuropsychological patients (e.g., Delis et al., 1986) and in studies using electroencephalography (e.g., Martens and Hubner, 2013) and functional imaging techniques (e.g., Fink et al., 1997).

Do people process visual information appearing near their left or right hands differently? In their study of cortically-blind participant MB, Brown et al. (2008) presented stimuli in the blind (upper-left) field and found that he was able to reliably indicate target size when he placed his left hand near the display (a configuration in which both visual field and hand are linked to the same RH), but not when he placed his right hand near the display. More recently, Tseng and Bridgeman (2011) found that participants performed a change-detection task more accurately when they placed both hands near the display in comparison to a no-hands condition, and also found that the right hand was somewhat more effective than the left hand in facilitating change detection. Tseng and Bridgeman concluded that facilitation in this change detection task was driven by a hand-related facilitation of visual working memory that reflects the frequency with which we commonly interact with objects. Le Bigot and Grosjean (2012) asked healthy right- and left-handed participants to perform an unspeeded visual discrimination task with the left hand, right hand, or both hands on the display, or no hands near the display. Both right- and left-handers demonstrated greater visual sensitivity near their dominant hand in comparison to their non-dominant hand. While right-handers did not show any benefit near their non-dominant left hand, left-handers did show some facilitation near their non-dominant right hand. Finally, Lloyd et al. (2010) showed greater effects of hand proximity in their target-discrimination task when the target appeared near the right hand. Importantly, Lloyd et al. avoided using the hands both as a manipulation and as an effector and instead asked participants to respond with their feet. Interestingly, they found that the right-hand proximity effect was significant only when participants responded with their right foot. Together, this set of studies indicates that the left and right hands may have differential effects on visual processing of nearby targets, but because these experiments did not explicitly test for interactions with cerebral lateralization, the following question remains unanswered.

Do the left and right hands have differential effects on the processing of nearby visual stimuli? The goal of this study was to test the hypothesis that presenting visual stimuli near the left hand preferentially recruits visual processing mechanisms lateralized in the RH, and also whether presenting visual stimuli near the right hand preferentially recruits visual processing mechanisms lateralized in the LH. To test this hypothesis, we capitalized on previous research showing that global and local visual information are processed preferentially in the right and LH, respectively (Sergent, 1982). Hierarchical letters were presented centrally and participants placed either their left or right hand nearby, or kept both hands far from the display. In an “attend-global” task, participants reported whether the target was present or absent at the global level as quickly as possible, and in an “attend-local” task, participants reported whether the target was present or absent at the local level as quickly as possible with their feet. We predicted that if visual stimuli appearing near the left hand preferentially recruit resources in the RH, then global-level processing should be facilitated in the left-hand present condition as compared to the right-hand present and hand-absent conditions. By contrast, if visual stimuli appearing near the right hand preferentially recruit resources in the LH, then local-level processing should be facilitated in the right-hand present condition as compared to the left-hand present and hand-absent conditions.

## Methods

### Participants

Thirty-one undergraduate students (mean age = 22.0 ± 6.64, range = 17–42) at Trent University participated in this study for extra credit or renumeration. All reported being strongly right-handed, with handedness scores greater than 28 on the Dutch Handedness Questionnaire (Van Strien, 1992). All had normal or corrected-to-normal visual acuity and no neurological history. The Trent University Research Ethics Board approved all procedures and each participant gave written informed consent before participation.

### Apparatus

**Figure 1:**
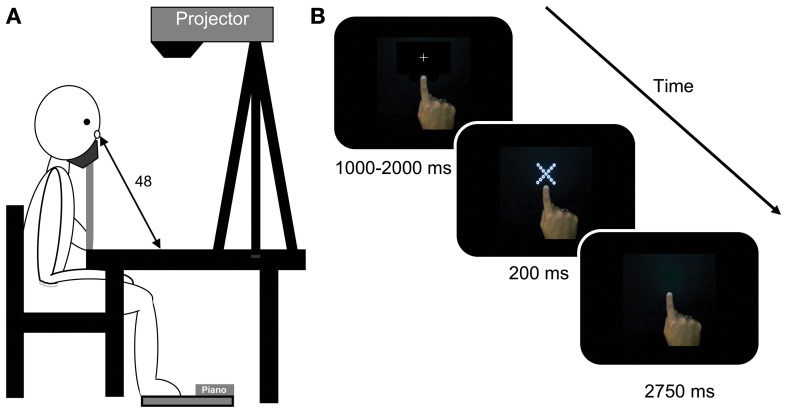
**(A)** The picture on the left depicts a participant sitting at the table where the display was projected by a projector mounted on a tripod. The participant responded by depressing the keyboard with his/her feet. **(B)** shows trial events in a typical right-hand near condition trial. The first screen was presented with the central fixation cross. The fixation was replaced by a hierarchical stimulus for 200 ms. This stimulus was removed and participants had up to 2750 ms to make their response. The trial ended and the next fixation was shown as soon as the response was made. Note: stimuli do not photograph as sharply as they appeared to participants.

Participants sat at a table and kept their head fixed in a chin rest with their feet resting on an electric piano (Yamaha PSR-270, Buena Park, CA) beneath the table (See Figure 1A). Displays were projected downward onto the table surface using an LCD projector (refresh rate = 75 Hz; Optoma DLP EP739, Mississauga, ON) onto a display space that was defined by a 66.0 × 50.8 cm sheet of matte black paper used to limit reflection. Displays were hierarchical forms (Table 1) created using GIMP (GNU Image Manipulation Program, The GIMP Development Team) and presented centrally in the display space in white against the black background of the experiment.

The experiment was programmed using the Psychophysics Toolbox extensions (Brainard, 1997; Pelli, 1997) for Matlab (The Mathworks™, Natick, MA). Response time (RT) data were collected on the same desktop computer receiving output from the electric piano placed at participants' feet. Participants made target-present/absent responses by pressing the piano keys with one foot or the other according to their assigned foot-response mapping. An algorithm in Matlab was used to detect the onset of the sound signal to determine RT. Matlab sampled the sound card directly at a rate of 16,384 Hz, and a Fourier transformation was used to determine whether the fundamental frequency of the signal was below or above a cutoff criterion. Low pitched tones represented a left-foot press and high-pitched tones represented a right-foot press. Pilot tests revealed 100% left-right classification accuracy.

### Displays

Displays consisted of hierarchical forms using small characters as local elements to compose large characters as the global elements (see Table 1). Target items were Xs or Os and non-target items were Ts and Us. These characters were chosen for their symmetry and similar proportion of straight (X and T) and curved (O and U) features, and all were presented in a sans serif font. Each display was 5 × 5 cm and spanned 6.0 degrees of visual angle (internal letters 0.9°) when presented at the mean viewing distance of 48 cm. Any given stimulus had a target located at both global and local levels, just the global level, just the local level, or no targets present at either level. All possible target configurations are presented in Table 1.

### Design

The experiment utilized a 2-task (attend global, attend local) × 3-hand (absent, left, right) × 2-target level (global vs. local) × 2-target presence (present, absent) within-subjects design. Each participant completed 2 versions of the target-detection task, which were blocked and counterbalanced across participants. In the attend-global task, participants were instructed to make their target-presence judgments on the large letter in the display and ignore the small component letters. In the attend-local task, participants were instructed to make their target-presence judgments on the identity of the small letters in the display while ignoring the large global letter. Hand was also blocked. Within each task level, participants completed 3 hand-level blocks: left-hand present, right-hand present, and hand-absent. Hand-level order was counterbalanced across participants. Target level was presented pseudorandomly throughout the experiment such that there were an equal number of stimuli with targets present and absent at the global and local levels in each task-condition (see Table 1 for target levels in experimental stimuli). At the beginning of each task, participants completed a 16-trial (hand-absent) practice block. Each of the 16 stimuli (Table 1) was presented three times per experimental block. Thus, the experiment consisted of six 48-trial blocks. Participants were provided with feedback about their speed (mean reaction time in ms) and accuracy (percent correct) after every 24 trials. Foot-response mapping was counterbalanced between participants such that half of the participants used the left foot for target-present responses and their right-foot for target-absent response, and the other half used the reverse mapping.

### Procedure

Participants were instructed on how to perform the first task condition with the aid of example stimuli that were chosen to demonstrate target presence and absence at the local and global levels (see Table 1). Participants were instructed about their assigned foot-response mapping and instructed to respond as quickly as they could while aiming for an accuracy rate of at least 90%. Participants completed a practice session for their assigned first task, and then completed the three experimental blocks for the assigned first task, one block for each of the three hand conditions. This process was then repeated for the second task condition.

For the left- and right-hand conditions, participant were asked to make a pointing posture with the hand of interest and place their index finger on a position marker presented 2 cm below the stimulus at the start of each experimental block. Participants kept their hand in this position and posture for the duration of experimental trials (see Figure 1B) while keeping their other hand away from the display by resting it on their lap. In the hand-absent condition participants were asked to keep both hands resting on their lap.

Each trial began with the presentation of a fixation cross in the centre of the display for a random duration between 1000 and 2000 ms. The display was then presented for 200 ms followed by a blank screen. Participants had a further 2750 ms to make their response (see Figure 1B). The experiment lasted for approximately 45 min.

## Results

Reaction time (RT) (ms) was recorded as participants made target-present/absent judgments about displays. The percentage of correct responses was calculated to measure each participant's performance accuracy. Before performing our statistical analyses, the following steps were taken. Participants whose overall accuracy rating failed to reach 90% were eliminated from the analysis. This resulted in the removal of two participants, leaving 29 participants' data for analysis. Trials in which participants did not respond were excluded from the analysis, resulting in the removal of 0.08% of the data. RT outliers were identified using the following rules. RTs lower than 100 ms were removed as research shows that participants need at least 90 ms to respond to newly-presented visual information (Paulignan et al., 1991). The overall mean and standard deviation of reaction time (ms) were determined from the remaining data and RTs greater than the mean plus four standard deviations (1500 ms) were removed, resulting in the loss of 0.10% of the data. Overall mean accuracy was 96.2 ± 2%. The arcsine transformation of proportion correct values within each cell of the design for each participant was calculated and these values used to analyse accuracy (Cohen and Cohen, 1983; Dixon, 2008). Incorrect responses were removed before mean reaction time for each cell of the design for each participant was calculated. These means were used to analyse reaction time.

### Global processing is facilitated by left- but not right-hand presence

**Figure 2:**
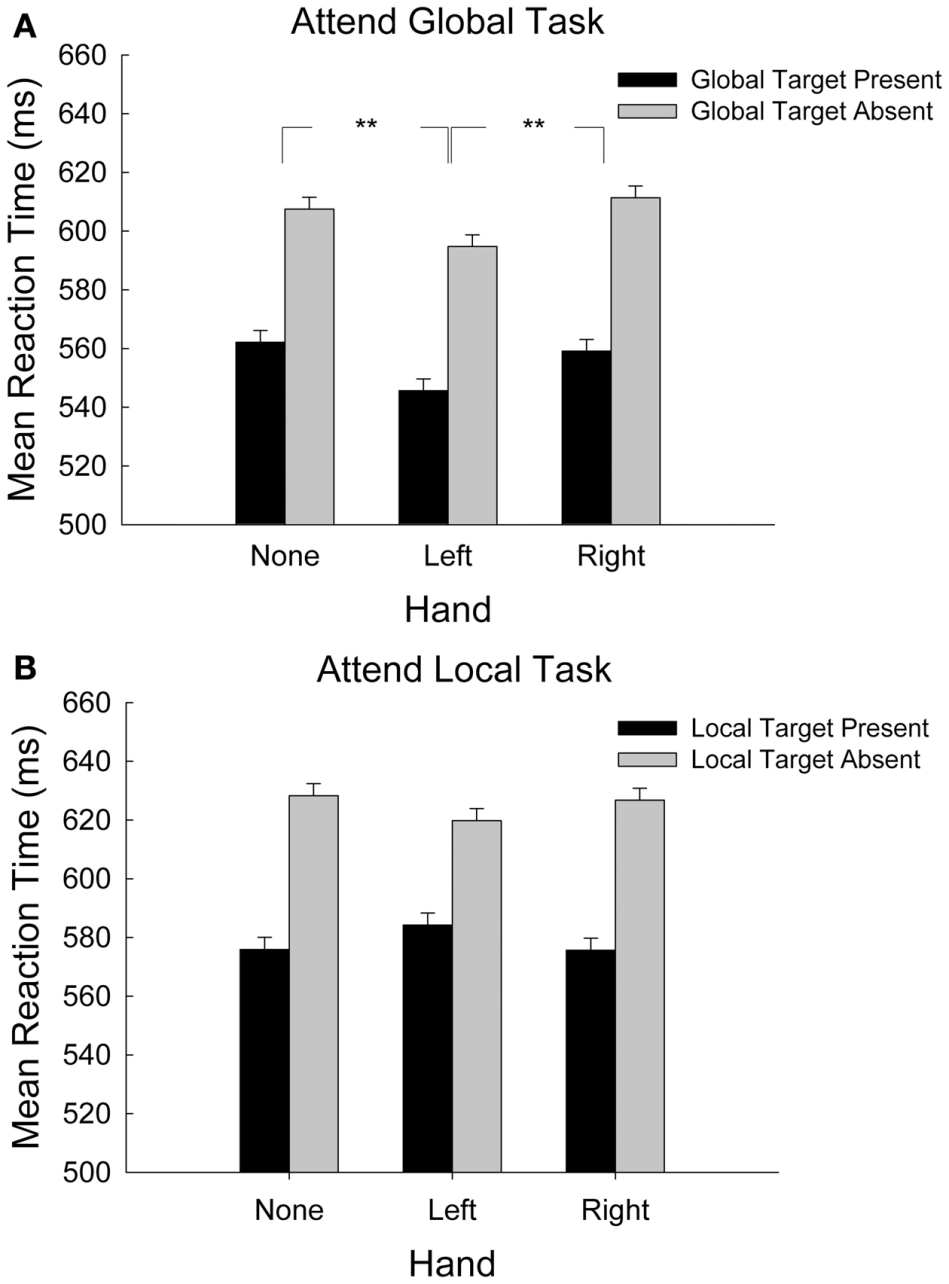
**(A)** Mean response time during the attend-global task, where global targets were present or absent, plotted by hand-presence condition. Error bars represent standard error of the mean. **(B)** Mean response time during the attend-local task, where local targets were present or absent, plotted by hand-presence condition. Error bars represent standard error of the mean ^**^p < 0.001.

Figure 2A shows mean reaction time as a function of hand condition and global target presence in the attend-global task only. We hypothesized that because global processing is linked to the RH, and because sensory processing for the left hand is also linked to the RH, placing the left hand near the display would recruit RH resources that would facilitate global processing. This hypothesis predicted that global target discrimination would be faster when stimuli were presented near the left hand than in the right-hand or no-hand conditions. We coded target presence according to whether the target was present or absent at the global level and then submitted RT data for correct responses to a 3-hand (left, right, absent) × 2-target presence (global-target present, global-target absent) repeated-measures analysis of variance (ANOVA, α = 0.05). We found significant main effects of target presence, *F*_(1, 28)_ = 55.75, *p* < 0.001, hand, *F*_(2, 56)_ = 3.59, *p* = 0.033, and no significant interaction, *F*_(2, 56)_ = 0.21, *p* = 0.881. Regarding the main effect for target presence, participants responded 48 ± 2 ms more quickly to global target-present stimuli than to target-absent stimuli. To determine the nature of the hand-presence effect we conducted planned comparisons (least significant difference (LSD), df = 28, α = 0.05) of mean RT for the three hand conditions. Responses were 20 ± 4 and 18 ± 4 ms faster with the left hand in the display in comparison to the right-hand and no-hand conditions, respectively (*p*s < 0.001). The right hand did not differ from the hand-absent condition (1.6 ± 4 ms), *p* = 0.917. Participants performed the global target-detection task more quickly when their left hand was in the display than when no hand or their right hand was present.

Although we eliminated participants who failed to achieve 90% correct overall, there remained a small possibility that the effect of hand on reaction time came at the expense of a shifted criterion for accuracy. To check for this possibility, we submitted the arcsine transformation of mean percent correct to the same ANOVA. This analysis revealed no significant effect of hand (*p* = 0.560), global target presence (*p* = 0.438), and no interaction (*p* = 0.242). Overall, participants completed the task with 96.4 ± 0.3% accuracy and the evidence suggests that they did not trade accuracy for speed when performing this task.

### Local processing is not sensitive to hand-presence

Figure 2B shows mean reaction time as a function of hand condition and local target presence in the attend-local task only. We hypothesized that because local processing is carried out predominantly in the LH, placing the right hand near the display would recruit LH resources that would facilitate local processing. This hypothesis predicted that local target discrimination would be faster when stimuli were presented near the right hand than in the left-hand or no-hand conditions. We coded target presence according to whether the target was present or absent at the local level and then submitted mean RT data for correct responses to a 3-hand (left, right, absent) x 2-target presence (local-target present, local-target absent) repeated-measures ANOVA. This analysis revealed a significant main effect of local target presence, *F*_(1, 28)_ = 34.78, *p* < 0.001. Mean reaction times for local target-present items were 46 ± 3 ms faster than for target-absent items. There was no significant main effect of hand, *F*_(2, 56)_ = 0.070, *p* = 0.932, nor was there a significant interaction between hand and local target presence, *F*_(2, 56)_ = 2.57, *p* = 0.086. Contrary to our predictions, placing the right hand near the display did not influence local processing^1^.

Our analysis of accuracy revealed no significant effect of hand (*p* = 0.685), global target presence (*p* = 0.137), and no interaction (*p* = 0.808). Overall, participants completed the task with 96.0 ± 0.3% accuracy and the evidence suggests that they did not trade accuracy for speed when performing this task.

### Global processing compared to local processing.

To investigate effects of task and to determine if response foot influenced the speed with which participants responded to the displays, we submitted mean RT for correct responses only to a 2-foot (left, right) × 2-task (attend-global, attend-local) × 3-hand (left, right, absent) × 4-target level (double target, single global target, single local target, no target) mixed ANOVA. This analysis revealed a main effect of task *F*_(1, 28)_ = 11.80, *p* = 0.002; participants performed the the attend-global task (576 ± 16 ms) significantly faster than the attend-local task (603 ± 12 ms). The ANOVA also revealed a task by target interaction, *F*_(3, 84)_ = 29.18, *p* < 0.001. Simple main effects analyses showed that when participants attended globally, there was no significant difference between responses to target-absent displays (602 ± 16 ms) and single local target displays (601 ± 16 ms; *p* = 0.892), indicating that when participants attended to the global level, they were not distracted by the presence of a target at the local level (*p* = 0.892). By contrast, when participants attended locally, they discarded target-absent displays (615 ± 12) significantly more quickly than they discarded displays with a target at the global level, *F*_(3, 84)_ = 55.07, *p* < 0.001. These results are reflective of the global precedence effect (Navon, 1977).

Consistent with the analyses reported earlier, this ANOVA revealed a marginal interaction of task and hand, *F*_(2, 56)_ = 2.73, *p* = 0.073. Planned analyses based on our predictions revealed that in the attend-global task, there was a significant effect of hand, *F*_(2, 56)_ = 3.89, *p* = 0.026. Comparisons between means (LSD, *df* = 28, α = 0.05) indicate that participants responded significantly more quickly when their left hand was placed near the display (563 ± 16) than in the right hand (583 ± 15 ms; *p* = 0.032) or no hand (581 ± 16 ms; *p* = 0.003) conditions. There was no difference between the left and right hands (*p* = 0.901). By contrast, in the attend-local task, no difference between hand conditions was revealed, *F*_(2, 56)_ = 0.01, *p* = 0.991. There were no other main effects or interactions involving hand.

Finally, this analysis revealed no significant main effect of foot, *F*_(1, 28)_ = 1.78, *p* = 0.19, and no significant interactions between foot and hand, *F*_(2, 56)_ = 0.12, *p* = 0.890, task, foot, and hand, *F*_(2, 56)_ = 0.08, *p* = 0.923, foot, target, and hand, *F*_(3, 84)_ = 1.75, *p* = 0.113, or foot, hand, task, and target, *F*_(6, 168)_ = 1.28, *p* = 0.268, indicating that response side (foot) did not influence the effect of the hand. There was a significant main effect of target, *F*_(3, 84)_ = 81.32, *p* < 0.001, and a significant interaction between foot and target, *F*_(3, 84)_ = 8.59, *p* < 0.001. Participants responded to double stimuli (550 ± 15 ms) significantly more quickly than to all other stimuli [global level alone (600 ± 14 ms); local level alone (599 ± 14 ms); no-target stimuli (608 ± 14 ms; all *p*s <.04)]. The interaction with foot was driven by the finding that differences between double-level targets and other targets were greater for the right (107 ± 16 ms) than left foot (36 ± 18 ms).

When the same 4-way ANOVA was applied to measures of response accuracy, a significant interaction of hand and foot was revealed, *F*_(2, 56)_ = 4.16, *p* = 0.021. Curiously, when there was no hand in the display, there was no difference in the accuracy of responses made by the right (96.5 ± 0.7%) and left (95.7 ± 0.7%) feet. Simple main effects analyses revealed, however, that when either hand was present in the display, left foot accuracy (97.3 ± 1.0%) was significantly better than right foot accuracy (94.3 ± 1.0%). This effect did not interact with task, *F*_(2, 56)_ = 0.397, *p* = 0.674, or with target type, *F*_(3, 84)_ = 0.118, *p* = 0.889. Importantly, there was no task by hand interaction, *F*_(2, 56)_ = 0.230, *p* = 0.795 indicating that participants did not trade speed for accuracy in this task.

### Are global items processed before local items?

**Figure 3:**
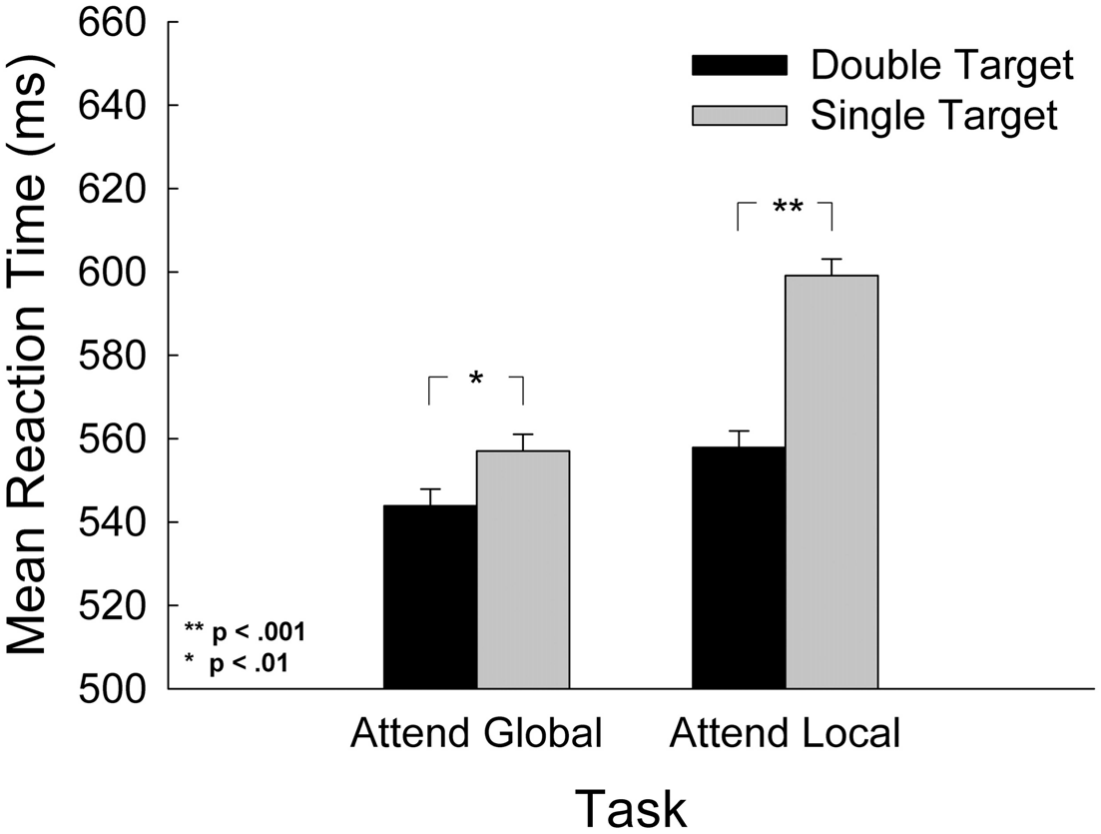
**Mean response time as a function of task and of target level, where double target contained a target at both the global and local levels, and single targets contained a target at the attended level only**. Error bars represent standard error of the mean.

To determine whether our stimuli assessed global and local processing in the manner we claim and in a manner consistent with past research, we checked our manipulation with the following analysis. According to Navon (1977) and Gestalt psychologists before him, global processing takes less time than local processing because humans are obligated to determine the global percept first. Alternative accounts of global precedence highlight the possibility that it may simply be easier to direct attention to the global, low-frequency stimulus level than the local, high-frequency stimulus level (e.g., Miller, 1981; Kimchi, 1982). Regardless, to assess whether this expected outcome was present in this study, we compared responses to displays with double targets (target present at both global and local levels) to those with a single target (target present at the attended level only) within each task. We also assessed the role that the nearby hand might play in the global precedence effect. We submitted mean RT to a 2-task (attend-global, attend-local) × 3-hand (left, right, absent) by 2-target type [double targets (target present at both the global and local level), single targets (targets present at the attended level only)] repeated measures ANOVA. The results are presented in Figure 3. The analysis revealed a significant interaction of task and target type, *F*_(1, 28)_ = 7.73, *p* = 0.009, and significant main effects for both task, *F*_(1, 28)_ = 13.38, *p* = 0.001, and target type, *F*_(1, 28)_ = 131.69, *p* < 0.001. For the attend-global task, participants responded to single target stimuli 37 ± 2 ms more slowly than double-target stimuli, and in the attend-local task, participants responded to single-target stimuli 61 ± 3 ms more slowly than to double-target stimuli. A simple main effects analysis revealed that the interference induced by non-targets at the unattended level (in single target stimuli) was significantly greater in the attend-local task than the attend-global task, *F*_(1, 28)_ = 7.81, *p* = 0.009.

This analysis also revealed a marginal interaction of task and hand, *F*_(2, 56)_ = 2.55, *p* = 0.087. Planned comparisons revealed that in the attend-global task, there was a significant main effect of hand, *F*_(2, 56)_ = 4.92, *p* = 0.011. Comparisons between means (LSD, df = 28) revealed that participants responded 19 ± 3 ms more quickly when the left hand was in the display in comparison to no hand (*p* < 0.001) and 19 ± 4 ms more quickly in comparison to the right hand (*p* = 0.005). There were no significant differences between hand conditions in the attend-local task, *F*_(2, 56)_ = 0.222, *p* = 0.802. There was no interaction between hand and target type. In sum, there is a greater cost for detecting single targets at the local level than at the global level. This finding is consistent with the long-standing global precedence effect and is an indicator that our stimuli adequately tapped global/low-frequency and local/high-frequency processing.

## Discussion

The purpose of the present study was to investigate the possibility that hand laterality and hemispheric lateralization of visual function interact to produce differential visual processing advantages near the left and right hands. We presented hierarchical forms near participants' left or right hands as well as in a hand-absent condition to investigate possible interactions with global and local processing, which have been linked to processing in the right and LHs, respectively. We predicted that global visual processing would be facilitated by placing the left hand near the stimulus, and that local processing would be facilitated by placing the right hand near the stimulus. We found that left-hand presence significantly improved the speed of discrimination of global-level targets in comparison to right-hand and hand-absent conditions. This improvement in global processing near the left hand was not achieved by compromising accuracy. This result is consistent with the possibility that the presentation of the target near the left hand preferentially recruited processing resources associated with the RH. We did not find that right-hand presence influenced local processing. Explanations for this pattern are presented below.

### Global processing facilitated by presenting hierarchical stimuli near the left hand

Participants discriminated global-level targets more quickly when their left hand was present near the display in comparison to right-hand present or hand-absent conditions. One possible explanation for this finding is that the presentation of the stimulus near the hands recruited visual-tactile bimodal cells linked to the hand in the contralateral RH (Graziano et al., 1994; Graziano, 1999) and that this recruitment preferentially facilitated right-hemisphere-dominant visual processing. Electrophysiological studies in monkeys have shown that these neurons, recorded in the hemisphere contralateral to the hand, have tRFs on the skin and vRFs that include and extend beyond the tRF into the space surrounding the hand. They are activated in response to either a tactile or a visual stimulus presented on or near the skin (Graziano and Gross, 1993; Graziano et al., 1994; Graziano, 1999; Graziano and Gandhi, 2000; Graziano and Cooke, 2006). It may be that presenting the hierarchical stimulus near the hand recruited bimodal neurons in the contralateral hemisphere and that they contributed to the visual processing of the stimulus. When the left-hand was placed near the stimulus, this recruitment facilitated global processing for two reasons. First, and most importantly, global processing was facilitated because both global processing and the sensory representation of the left hand are linked strongly to the RH. Second, global processing precedes local processing (Navon, 1977), unfolding relatively early in the stream of visual processing. Evidence suggests that near-hand effects influence relatively early aspects of perception, like figure-ground segregation (Cosman and Vecera, 2010). More recently, research has demonstrated that hand presence may preferentially activate the temporally-sensitive magnocellular visual pathway while inhibiting the spatially-sensitive parvocellular visual pathway (Gozli et al., 2012). Therefore, one possibility is that the observation of left-hand facilitation of global processing depends both on (1) the congruency between left-hand sensory processing and global processing dominance in the RH, and (2) the notion that both hand-presence and the grouping mechanisms that give rise to the global percept act relatively early in the stream of visual processing (Pomerantz and Pristach, 1989; Moore and Egeth, 1997; Gozli et al., 2012).

### Local processing of hierarchical stimuli did not benefit from near-hand presence

Hand presence near the display did not influence either the speed or accuracy with which participants discriminated targets at the local level. It is possible that the current task failed to demonstrate clear near-hand effects for local processing because hand effects happen relatively early in the stream of visual processing (Cosman and Vecera, 2010) whereas local processing happens later (Navon, 1977). Our analysis of the global precedence effect indicates that people have more difficulty discarding a global distractor than a local distractor. Our hand condition did not interact with these effects, which is somewhat inconsistent with the findings of Davoli et al. (2012) who found that switching attention between global and local levels was delayed by the presence of two hands near the display. There are several key differences between their study and this one, however, that may explain this inconsistency. First, we did not have a two-hand condition in our experiment, and second, Davoli et al. (2012) asked their participants to not only switch from a global identification task to a local identification task within one trial, but they also asked their participants to switch from one stimulus to another. These differences make the two experiments very difficult to compare. In general, we believe that these attend-local findings are consistent with the proposal that global processing happens early and is obligatory (Navon, 1977; Conci et al., 2011), whereas local processing happens later and may not be obligatory. Since local information is dealt with later, it may be more difficult to isolate hand effects on local processing using response time measures.

### Possible explanations for near-hand effects

Explanations for near-hand effects have been offered both at cognitive and neural levels and it is important to understand these effects at both levels. In general, cognitive-level accounts (Reed et al., 2006, 2010; Abrams et al., 2008; Cosman and Vecera, 2010; Tseng and Bridgeman, 2011; Davoli et al., 2012), have focused on explaining the conditions that invoke facilitation vs. interference and often examine the effects of placing both hands near the display. In general, cognitive-level explanations have been compatible with neural-level explanations.

One possible explanation is that improved visual processing of targets appearing near a passively resting hand is simply an epiphenomenon of the roles that sensory and motor systems play in covert preparation for action (Reed et al., 2010; Gozli et al., 2012; Makin et al., 2012). Objects presented near the hands are often associated with actions and these potential actions demand effective coding of stimulus location with respect to our limbs so that we can interact with our environment efficiently. For example, during a reaching action, grip and/or trajectory adjustments may be needed to improve the movement's completion or respond to unexpected target motion. As such, the activation of bimodal cells by near-hand targets may work to represent the target in a hand-centred frame of reference that is better prepared to initiate new actions or adjust ongoing ones, if need be (Reed et al., 2010; Makin et al., 2012). Bimodal cells may also play a role in acting quickly on visual targets that appear suddenly within peripersonal space (Graziano and Cooke, 2006).

While this possibility requires further testing, it does not appear to explain the data we present here. Evidence suggests that visual processing for reaching and grasping is lateralized to the LH, even in left-handers (Gonzalez et al., 2006). This lateralization predicts that, regardless of the task participants were performing, if participants were covertly preparing to grasp our hierarchical stimuli, we should have observed an effect of placing the *right* hand near the stimuli. The effect we present here is clearly linked to the near left hand.

We believe that the differential effect of the nearby left and right hand on global processing described here can be explained by a bimodal-recruitment model that takes into account the lateralized sensory processing associated with each hand. When a target appears near a hand, bimodal cells are recruited to help process the target, whereas when the hand is not nearby the target, these cells are not recruited. We propose that the additional activation of bimodal cells in the near-hand case improves the representation of the target. The near-hand visual representation of the target is more robust, more resolute, and therefore, responses can be made earlier and with less variability. This explanation is like the one used to explain redundancy gains (e.g., Raab, 1962; Gielen et al., 1983). Redundancy gains are explained by the notion that two identical stimuli recruit more resources than one stimulus, and that these resources either combine or compete for response activation, leading to better performance in the two-stimulus condition (e.g., Mordkoff and Yantis, 1991; Mordkoff and Miller, 1993). Support for this explanation of near-hand effects can be derived from previous studies showing reductions in the variability of size-estimation and grasping (Brown et al., 2008), targeted-reaching performance (Brown et al., 2009), and improvements in signal sensitivity (Dufour and Touzalin, 2008; Le Bigot and Grosjean, 2012) in near-hand conditions. Together, these findings suggest that reductions in variability reflect reductions in noise as additional (bimodal) neurons are recruited for processing. Thus, presenting targets near the hand may result in an overall improvement in the signal-to-noise ratio (SNR).

An additional part of this explanation relies on the possibility that bimodal-cell recruitment is lateralized to the hemisphere contralateral to the hand of interest. Because tactile responses are highly lateralized to the hemisphere contralateral to the hand (Bryden, 1982; Graziano, 1999; Jones and Lederman, 2006; Reed et al., 2009), we surmise that bimodal cell responses are lateralized in a similar manner. Functional imaging studies are consistent with this idea. All reports of brain activation to visual targets appearing near a hand primarily show activation in the hemisphere contralateral to the hand (Makin et al., 2007; Brozzoli et al., 2011; Gentile et al., 2011). If the activation of bimodal cells in response to visual stimuli appearing near the hand is largely confined to the contralateral hemisphere, then we propose that this activation will have preferential access to any specialized visual processing happening there. Our finding that global processing, a preferentially right-hemisphere function, benefits from having the left but not the right hand near the target is consistent with this notion.

While this possibility also requires further testing, a computation model developed on the basis of similar assumptions has had success reproducing near-hand (Magosso et al., 2010a) and near-tool effects (Magosso et al., 2010b) in humans. The model assumes that the left and RHs initially code space near the left and right hands independently, and that interactions between hemispheres happen after a near-hand stimulus has been coded by a visual system, a tactile system, and then by a downstream visual-tactile system that integrates visual and tactile information from space near and on the hands. Tests of the model have revealed that this relatively simple architecture can reproduce effects demonstrated in studies of humans, including the reinforcement of unisensory perception by multimodal activation.

## Conclusion

In short, the nature of near-hand effects may rely both on which hand appears near the display and hemispheric specialization: stimuli appearing near a hand may recruit bimodal visual-tactile neurons in the contralateral hemisphere, stimulating lateralized visual processing mechanisms there. The data we present here provide partial support for this hypothesis.

## Author contributions

Liana E. Brown, Robin M. Langerak, and Carina La Mantia conception and design of research; Robin M. Langerak performed experiments; Robin M. Langerak and Liana E. Brown analyzed data; Robin M. Langerak and Liana E. Brown interpreted results of experiments; Robin M. Langerak and Liana E. Brown prepared figures; Robin M. Langerak, Carina La Mantia and Liana E. Brown drafted manuscript.

### Conflict of interest statement

The authors declare that the research was conducted in the absence of any commercial or financial relationships that could be construed as a potential conflict of interest.

## Footnotes

^1^Although there is a pattern in the attend-local means suggesting that the left hand may interfere with “yes” responses to local target-present displays and facilitate “no” responses to local target-absent displays, an analysis of simple main effects of hand within each target level revealed no significant differences between hand presence in either the target present [*F*_(2, 56)_ = 0.59, *p* = 0.557] or target-absent [*F*_(2, 56)_ = 0.76, *p* = 0.474] conditions.
